# Pacing of Women and Men in Half-Marathon and Marathon Races

**DOI:** 10.3390/medicina55010014

**Published:** 2019-01-14

**Authors:** Pantelis T. Nikolaidis, Ivan Ćuk, Beat Knechtle

**Affiliations:** 1 Exercise Physiology Laboratory, 18450 Nikaia, Greece; 2 Faculty of Physical Education and Sports Management, Singidunum University, 11000 Belgrade, Serbia; icuk@singidunum.ac.rs; 3 Institute of Primary Care, University of Zurich, 8006 Zürich, Switzerland; beat.knechtle@hispeed.ch

**Keywords:** aerobic exercise, endurance, marathon, performance, running

## Abstract

*Background and objective*: Half-marathon is the most popular endurance running race in terms of number of races and runners competing annually; however, no study has compared pacing strategies for this race distance with marathon. The aim of the present study was to profile pacing in half-marathon, compare half-marathon and marathon for pacing, and estimate sex differences in pacing. *Materials and methods*: A total of 9137 finishers in the half-marathon (*n* = 7258) and marathon race (*n* = 1853) in Ljubljana 2017 were considered for their pacing in five race segments (0–23.7%, 23.7–47.4%, 47.4–71.1%, 71.1–94.8%, and 94.8–100% of the race. *Results*: Half-marathon runners followed a positive pacing with every segment being slower than its previous one without the presence of an endspurt. Compared to marathon (where the average percent of change in speed (ACS) was 5.71%), a more even pacing was observed in half-marathon (ACS = 4.10%). Moreover, women (ACS = 4.11%) had similar pacing as men (ACS = 4.09%) in half-marathons. *Conclusions*: In summary, running a half-marathon followed a unique pattern that differentiated this race distance from marathon, with the former showing a more even pacing with an absence of endspurt, and sex difference compared to the latter. Consequently, runners should be advised to adopt a less variable pacing when competing in a half-marathon, regardless of their sex. To the best of our knowledge, the more even pacing in half-marathon, than in marathon, was a novel finding, as it was the first study to compare the two race distances for this characteristic.

## 1. Introduction

The half-marathon has evolved as a sport discipline of increasing popularity, documented by the annual number of finishers and races taking place worldwide [1]. In general, performance in endurance running, as well as in endurance sports of other modes of exercise (cycling, swimming, cross-country skiing), has been shown to be associated with pacing, among other physiological and psychological variables [2,3]. Despite the popularity of half-marathon, limited information about the pacing, in this sport discipline, exists [4]. Since half-marathon has been characterized by an increased woman participation compared to marathon [5], and sex differences in pacing in marathon have been observed [2,3], estimating the pacing in half-marathon would be of great practical interest, especially considering the aspect of sex.

Pacing has been well studied in many endurance and ultra-endurance sports, such as cycling [6,7], swimming [8,9], and triathlon [10]. It has been observed that pacing in cycling varied depending on whether cyclists performed exercise for a given time or distance, showing a faster start when competing for distance [6]. Research on the world’s longest ultra-cycling race showed a decrease of speed across the race [7]. The variation of speed, in 800 m for women [8] and 1500 m freestyle swimming for men, followed a U shape [9]. In sprint triathlon, a comparison among three pacing strategies (positive, negative, and even, where speed either decreased, increased, or remained stable across race) of swimming indicated that a positive pacing in swimming induced a lower rate of perceived exertion than a negative pacing [10]. The abovementioned studies found different pacing strategies among endurance and ultra-endurance sports, which did not allow the generalization of their findings to other sports. Adopting a pacing strategy might depend on athletes’ decision making [11], perception of risk [12], and individual variability [13]. For instance, pacing might be influenced by internal bodily state feedback, teleoanticipation, template formation of perceived exertion, and human-environment interaction (e.g., interaction among competitors) [11]. In addition, it was shown that cyclists and ultra-marathon runners with low perception of risk started the race faster than their counterparts with high perception of risk [12]. Furthermore, inter-individual differences in the distribution of effort, across a race, might be partially explained by athletes’ motivation [13].

So far, with regards to endurance running sport disciplines of high popularity, pacing has been well studied in marathon, where it has been found that runners adopted a positive pacing, i.e., their speed decreased across race, accompanied by the presence of an end spurt, i.e., the speed increased in the final section [2,14,15,16]. A positive pacing has also been observed in shorter distances, such as 800 m [17], 5 km [18], and 10 km [19]. With regards to sex differences, women marathon runners adopted a more even pacing in marathon [3,20] and in 100 km, than men [21]. Perceived effort and physiological parameters have been identified as correlates of pacing, and their role might vary across a 10 km race; e.g., perceived effort influenced speed at the start of the race, whereas mainly aerobic capacity and muscle strength-to a lesser degree-influenced speed for the rest of the race [22]. Moreover, with regards to the relationship of pacing with motivation, it has been shown that men with more even pacing scored higher in psychological coping, self-esteem, life meaning, recognition, and competition, than their counterparts with more variable pacing [23].

Although many studies have been conducted on the pacing of marathon [15,16,24,25,26], the limited relevant information that existed for half-marathon [4] had focused on elite runners. Since these two distance races differed for performance characteristics [5], it would be reasonable to assume that knowledge from pacing in marathon could not be “transferred” to half-marathon. Knowledge about pacing in half-marathon would have both theoretical and practical interest. From a theoretical perspective, exercise physiologists would be interested in the patterns of energy distribution across a 21 km running race and on potential sex differences in these patterns. From a practical perspective, coaches and fitness trainers working with half-marathon runners would use such knowledge to assist their athletes adopting sex-tailored pacing strategies. Therefore, the aim of the present study was to (a) examine changes in speed and whether pacing would be positive, negative, or even across half-marathon and marathon races, (b) investigate whether pacing would vary by race distance, and (c) estimate sex differences in pacing for both race distances. It was hypothesized that half-marathon would present a positive pacing with an endspurt, and women would adopt a more even pacing than men.

## 2. Materials and Methods

### 2.1. Participants

This study was approved by the Institutional Review Board of Kanton St. Gallen, Switzerland, with a waiver of the requirement for informed consent of the participants as the study involved the analysis of publicly available data. The study was conducted in accordance with recognized ethical standards according to the Declaration of Helsinki adopted in 1964, and revised in 2013.

For the purpose of this study, valid results and split times from 1853 participants (age 41.7 ± 9.8 years, range 17–78 years; average race speed 3.03 ± 0.46 m/s, 2.13–5.47 m/s; mean ± standard deviation) of the 2017 Ljubljana marathon and 7258 participants (age 40.3 ± 10.7 years, 12–86 years; 2.99 ± 0.45 m/s, 1.61–5.15 m/s) of the 2017 Ljubljana half-marathon (total 9137 participants) were analyzed. The obtained data presents publicly available, official results from the “Ljubljana Marathon” website [27]. Participants who did not finish the race, or did not have any recorded split times, were excluded from the study. Both marathon and half-marathon were held on the same day and on the same track, whereas half-marathon race is entirely contained within marathon race. This approach assisted in eliminating the potential influence of environmental conditions [28]. Moreover, both marathon and half-marathon were rather flat, with an elevation difference of 29 m (ranging from 295–324 m). The temperature on the race day ranged from 4.2–15.4 °C, without strong wind or excess humidity.

### 2.2. Variables and Research Measures

From the available data, the average race speed 0–100% (0–21.0975 km for half-marathon and 0–42.195 km for marathon) was calculated. Moreover, average running speed in five race segments was estimated for both marathon and half-marathon, that corresponded to:

Segment 1-Average running speed from 0–23.7% of the race (i.e., 0–5 km for half-marathon and 0–10 km for marathon)
Segment 2-Average running speed from 23.7–47.4% of the race (i.e., 5–10 km for half-marathon and 10–20 km for marathon)
Segment 3-Average running speed from 47.4–71.1% of the race (i.e., 10–15 km for half-marathon and 20–30 km for marathon)
Segment 4-Average running speed from 71.1–94.8% of the race (i.e., 15–20 km for half-marathon and 30–40 km for marathon)
Segment 5-Average running speed from 94.8–100% of the race (i.e., 20–21.0975 km for half-marathon and 40–42.195 km for marathon)

### 2.3. Process

Thereafter, the percentage of average change in speed for each segment (ACSS), with regards to the average race speed, was calculated. The applied equation is as follows: ACSS = 100 − (100 × average race speed/average segment speed). Finally, the average percentage of change in speed, through the 5 race segments (average change in speed (ACS)), was estimated. Note that absolute values of ACS were presented and statistically tested. The applied equation was as follows: ACS = (ACSS1 + ACSS2 + ACSS3 + ACSS4 + ACSS5)/5. Changes in speed across a running race have been previously used to study pacing in 800 m [17], 5 km [18], 10 km [19], half-marathon [4], and marathon [15]. In the present study, the speed in each segment was expressed as the percentage difference from the average race speed, in order to provide comparable data considering potential differences in average race speed between races or sexes.

### 2.4. Data Analysis

Prior to all statistical tests, descriptive statistics were calculated as mean, standard deviation, and minimum and maximum values. Since the Kolmogorov-Smirnov test is not sensitive in large samples, data distribution normality was assessed by inspecting histograms and QQ plots. After careful examination, the obtained data showed rather normal distribution. Mixed between-within analysis of variance (ANOVA) was performed for ACSS to test differences between segments (i.e., Segments 1–5; within-subject factor), race (i.e., marathon and half-marathon; between-subjects factor), as well as their interaction (segment × race). To further assess race differences, four additional mixed between-within ANOVAs for ACSS were performed. Two ANOVAs were performed to assess differences between segments (i.e., Segments 1 to 5; within-subject factor), race (i.e., marathon and half-marathon; between-subjects factor) as well as their interaction (segment × race), separately for men and women. Another two ANOVAs were performed to assess differences between segments (i.e., Segments 1 to 5; within-subject factor), sex (i.e., men and women), as well as their interaction (segment × sex), separately for marathon and half-marathon. Finally, one two-way ANOVA was performed on ACS to assess differences between races (i.e., marathon and half-marathon), sex (i.e., men and women), as well as their interaction (race × sex). For all ANOVAs, a post hoc Bonferroni test was performed. Effect size was presented via eta squared (ŋ^2^), where the values of 0.01, 0.06, and above 0.14 were considered small, medium, and large, respectively [29]. Alpha level was set at *p* < 0.05. All statistical tests were performed using Microsoft Office Excel 2007 (Microsoft Corporation, Redmond, WA, USA) and SPSS 20 (IBM, Armonk, NY, USA).

## 3. Results

**Figure 1:**
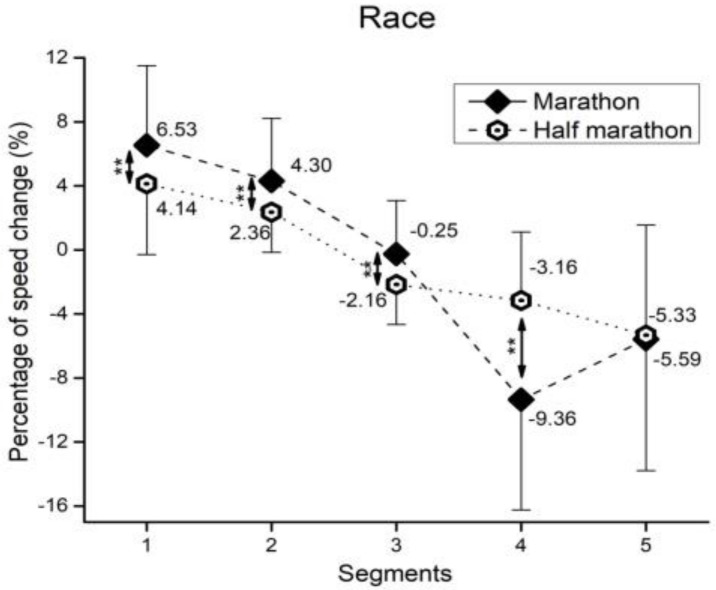
Percentage of speed change by split section in half-marathon and marathon. Error bars present standard deviation. ** *p* < 0.01 for significant difference between races.

**Figure 2:**
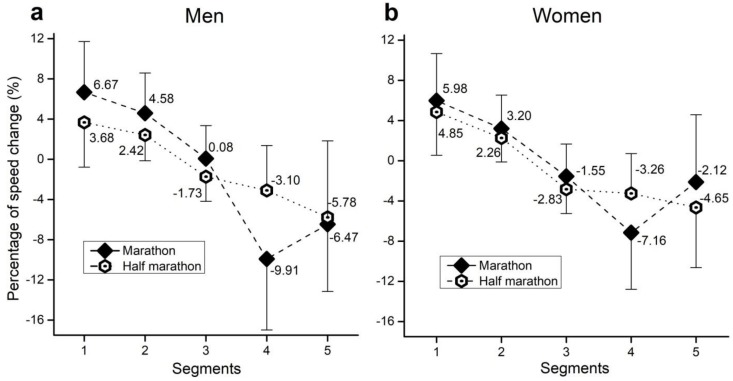
Percentage of speed change by segment and race distance in men (**a**) and women (**b**). Error bars present standard deviation.

**Figure 3:**
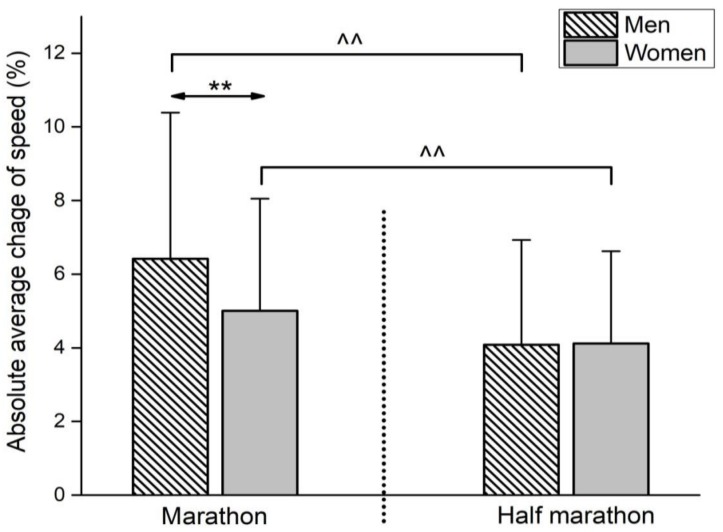
Absolute average change of speed by race distance and sex. Error bars present standard deviation. ** *p* < 0.01 for differences between sexes; ^^ *p* < 0.01 for differences between races.

The average running speeds for five segments, as well as the average race speed of participants by sex and race distance, are presented in Table 1. From the descriptive data in Table 1, a gradual decrease in average speed through the race segments was observed for both sexes in both marathon and half-marathon, e.g., from 3.26 ± 0.44 m/s in segment 1 to 2.99 ± 0.50 m/s in segment 5 in men half-marathon runners. Moreover, the largest deviation of running speed was observed in marathon men, i.e., decrease by 0.38 m/s from segment 1–5, whereas the smallest deviation of running speed was observed in half-marathon women, i.e., decrease by 0.25 m/s from segment 1–5. Further examination of participants’ speed and speed change is presented in Figure 1, Figure 2 and Figure 3.

In regards to marathon and half-marathon runners of both sexes (Figure 1), the significant main effects of segment (*F*(4, 9106) = 5959.2, ŋ^2^ = 0.36, large magnitude, *p* < 0.01), race (*F*(4, 9106) = 11.7, ŋ^2^ < 0.01, trivial, *p* = 0.17), and segment × race interaction (*F*(4, 9106) = 723.1, ŋ^2^ = 0.04, small, *p* < 0.01) were observed. Specifically, in both marathon and half-marathon runners, each segment significantly differs (*p* < 0.01) in speed change compared to the others.

When only men runners were considered (Figure 2a), results indicated the significant main effects of segment (*F*(4, 5879) = 4392.3, ŋ^2^ = 0.39, large, *p* < 0.01), race (*F*(4, 5879) = 64.2, ŋ^2^ < 0.01, trivial, *p* < 0.01), and segment × race interaction (*F*(4, 5879) = 609.4, ŋ^2^ = 0.05, small, *p* < 0.01). Specifically, for the first two segments (2.99% and 2.16% respectively), and the last two segments (6.81% and 0.69% respectively), men marathon runners showed greater changes in speed than half-marathon runners of the same sex (*p* < 0.01). However, in the third segment, men marathon runners presented more stable speed than half-marathon runners by 1.81% (*p* < 0.01).

Similar results were obtained when only women runners were observed (Figure 2b). Significant main effects of segment (*F*(4, 3222) = 1279.2, ŋ^2^ = 0.27, large, *p* < 0.01), race (*F*(4, 3222) = 57.3, ŋ^2^ < 0.01, trivial, *p* < 0.01), and segment × race interaction (*F*(4, 3222) = 100.0, ŋ^2^ = 0.02, small, *p* < 0.01) were observed. In particular, for the first two segments (1.13% and 0.94% respectively), and the fourth segment (3.90%), women marathon runners showed a greater speed change than women half-marathon runners (*p* < 0.01). On the other hand, in the third (1.28%) and the fifth segment (2.53%), women marathon runners exhibited more stable speed than women half-marathon runners of the same sex (*p* < 0.01).

When marathon runners only were observed (Figure 2a,b), the results confirmed the significant main effects of segment (*F*(4, 1848) = 1131.2, ŋ^2^ = 0.36, large, *p* < 0.01), sex (*F*(4, 1848) = 88.0, ŋ^2^ < 0.01, trivial, *p* < 0.01), and segment × sex interaction (*F*(4, 1848) = 57.0, ŋ^2^ = 0.02, small, *p* < 0.01). Specifically, for the first two segments (0.69% and 1.38% respectively), and the last two segments (2.75% and 4.35% respectively), men marathon runners presented greater changes in speed than women marathon runners (*p* < 0.01). For the third and fourth segments (1.63% respectively), men marathon runners showed more stable speed than women marathon runners (*p* < 0.01).

Considering half-marathon results only (Figure 2a,b), the significant main effects of segment (*F*(4, 7253) = 4772.6, ŋ^2^ = 0.38, large, *p* < 0.01), sex (*F*(4, 7253) = 46.9, ŋ^2^ < 0.01, trivial, *p* < 0.01), and segment × sex interaction (*F*(4, 7253) = 71.3, ŋ^2^ = 0.01, trivial, *p* < 0.01) were observed. Particularly, in the first three segments (1.17%, 1.10%, and 0.17%, respectively), men half-marathon runners had more stable speed than women (*p* < 0.01), whereas in the fifth segment (1.13%) women had more stable speed (*p* < 0.01). Regarding fourth segment (0.16%), no significant differences between men and women half-marathon runners were observed.

Finally, in men and women runners in both marathon and half-marathon, each segment significantly differences in speed change than the other (*p* < 0.01). The only exception is the lack of difference between the third and fifth segment in women marathon runners (*p* = 0.96).

Regarding ACS (Figure 3), the significant main effects of race (*F*(3, 9107) = 300.8, ŋ^2^ = 0.03, small, *p* < 0.01), sex (*F*(3, 9107) = 55.6, ŋ^2^ = 0.01, trivial, *p* < 0.01), and race × sex interaction (*F*(3, 9107) = 60.5, ŋ^2^ = 0.01, trivial, *p* < 0.01) were observed. Men marathon runners had a greater average change of speed of 2.34% than half-marathon runners (*p* < 0.01), whereas women marathon runners had a 0.89% greater change of speed than women half-marathon runners (*p* < 0.01). Moreover, the difference between men and women in marathon showed that there was a 1.41% greater average change of speed in marathon men (*p* < 0.01). Finally, the average speed difference between men and women in half-marathon was only 0.030% (*p* = 0.67).

## 4. Discussion

The main findings of the present study were that (a) half-marathon runners followed a positive pacing with every segment being slower than its previous one without the presence of an endspurt; (b) compared to marathon, a more even pacing was observed in half-marathon; and (c) women had similar pacing as men in half-marathon.

The overall distribution of energy across the race in the half-marathon followed the so-called “positive pacing” [30], that is, the speed decreased continuously across the race. This observation was in agreement with a previous research on IAAF World Half Marathon Championships where slower athletes had decreased speeds from the first segment onwards [4]. It should be highlighted that no endspurt was shown, which was in disagreement with the study of Hanley [4] who examined only elite runners. The discrepancy between the two studies might be attributed to the different performance level, as the abovementioned study focused on world championships, where the faster athletes showed larger endspurt than their slower peers. This suggested that lack of an endspurt might appear in half-marathon races with a large participation of recreational runners.

To the best of our knowledge, the more even pacing in half-marathon than in marathon was a novel finding, as it was the first study to compare the two race distances for this characteristic. It might be assumed that this difference was due to the additional fatigue induced in the marathon. This assumption is supported by the observation that their difference reached its peak in the fourth segment, i.e., close to the end of the race. On the other hand, both race distances adopted a positive pacing, which was in line with the notion that the perception of effort scaled with the proportion of exercise time that remained [31].

With regards to sex differences, surprisingly, women had similar pacing as men in a half-marathon. In marathon, women had a more even pacing than men, which confirmed the previous findings in other marathon races [20,25,26]. This sex difference has been attributed to differences in physiology and decision making between women and men [32].

Both half-marathon and marathon runners adopted a variable pacing instead of maintaining a steady speed across race. Following a variable self-pacing has been shown to present certain advantages-i.e., enhancement of critical power and high-intensity exercise performance compared to constant work rate cycling exercise [33]. In addition, the rate of perceived exertion has been shown to associate with pacing [22] and might vary across race [34]. Moreover, it was acknowledged that head-to-head competition improved performance compared to running alone [35]. Although this aspect was not examined in the present study, it would be assumed that head-to-head competition exerted a similar influence in both race distances, since half-marathon and marathon races were massive events. Overall, pacing should be considered as a complex system, where individual responses interacted with environment [36]. In this context, athletes were requested to balance behavior and thinking (self-regulation) to optimize their speed across the race [37].

A limitation of the present research was that it considered a sport event (Ljubljana) with relatively small participation compared to other races [38]. Thus, the findings should be considered as preliminary, and should be verified in future studies. On the other hand, strength was the novelty as it added original information in the existing literature with regards to one of the most popular race distances. Considering that half-marathon was the most popular running race event in terms of annual number of races and participants [1,5], the findings of the present study would have practical applications for a wide range of professionals working endurance runners, e.g., coaches, fitness trainers, nutritionists, and physicians. As it has been shown that endurance runners might compete to both half-marathon and marathon [39], it would be of great practical relevance to know how these two race distances differed in pacing. With regards to sex, men and women should be advised to adopt similar pacing patterns in half-marathon, whereas women should aim to run more evenly than men in marathon. Considering the race distance, runners should be guided to regulate their pacing as less or more variable, depending on whether they intended to run a half-marathon or marathon, respectively. Since recent studies reported an association of pacing with physiological and psychological parameters in marathon [23] and 10 km run [22], future research should verify this association in half-marathon, too.

## 5. Conclusions

In summary, both half-marathon and marathon races presented a positive pacing, i.e., speed decreased across the race; however, half-marathon runners did not show an endspurt, which was observed in marathon runners of the present study, as well as of all previous research in marathon. Furthermore, women and men adopted similar pacing pattern in half-marathon, whereas in marathon, women had more even pacing than men that was in agreement with the existing literature about marathon. Consequently, runners should be advised to adopt a less variable pacing when competing in a half-marathon, regardless of their sex. Further research would be needed to shed light on the physiological and psychological correlates of pacing in half-marathon.

## Figures and Tables

**Table 1:** Segments and race speed for men and women, marathon, and half-marathon runners.

		Segment 1 Speed (m/s)	Segment 2 Speed (m/s)	Segment 3 Speed (m/s)	Segment 4 Speed (m/s)	Segment 5 Speed (m/s)	Average Race Speed (m/s)
Men 42.2 km *N* = 1478	Median	3.24	3.18	3.06	2.80	2.88	3.04
	Mean	3.29	3.22	3.09	2.82	2.91	3.08
	SD	0.44	0.45	0.49	0.52	0.49	0.46
	CV	0.13	0.14	0.16	0.18	0.17	0.15
Women 42.2 km *N* = 375	Median	2.96	2.90	2.78	2.63	2.77	2.80
	Mean	3.04	2.95	2.82	2.68	2.81	2.86
	SD	0.36	0.38	0.43	0.44	0.41	0.39
	CV	0.12	0.13	0.15	0.16	0.15	0.14
Men 21.1 km *N* = 4406	Median	3.21	3.18	3.06	3.04	2.97	3.10
	Mean	3.26	3.22	3.09	3.06	2.99	3.14
	SD	0.44	0.44	0.46	0.49	0.50	0.45
	CV	0.14	0.14	0.15	0.16	0.17	0.14
Women 21.1 km *N* = 2852	Median	2.90	2.83	2.69	2.69	2.64	2.76
	Mean	2.91	2.83	2.70	2.69	2.66	2.77
	SD	0.32	0.35	0.36	0.39	0.39	0.35
	CV	0.11	0.12	0.14	0.14	0.15	0.13

SD = standard deviation; CV = coefficient of variation.

## References

[1] Knechtle B., Nikolaidis P.T., Onywera V.O., Zingg M.A., Rosemann T., Rust C.A. (2016). Male and female ethiopian and kenyan runners are the fastest and the youngest in both half and full marathon. SpringerPlus.

[2] Nikolaidis P.T., Knechtle B. (2017). Effect of age and performance on pacing of marathon runners. Open Access J. Sports Med..

[3] Breen D., Norris M., Healy R., Anderson R. (2018). Marathon pace control in masters athletes. Int. J. Sports Physiol. Perform..

[4] Hanley B. (2015). Pacing profiles and pack running at the IAAF world half marathon championships. J. Sports Sci..

[5] Knechtle B., Nikolaidis P.T., Zingg M.A., Rosemann T., Rust C.A. (2016). Half-marathoners are younger and slower than marathoners. SpringerPlus.

[6] Abbiss C.R., Thompson K.G., Lipski M., Meyer T., Skorski S. (2016). Difference in pacing between time- and distance-based time trials in trained cyclists. Int. J. Sports Physiol. Perform..

[7] Heidenfelder A., Rosemann T., Rust C.A., Knechtle B. (2016). Pacing strategies of ultracyclists in the “race across America”. Int. J. Sports Physiol. Perform..

[8] Lipinska P., Allen S.V., Hopkins W.G. (2016). Modeling parameters that characterize pacing of elite female 800-m freestyle swimmers. Eur. J. Sport Sci..

[9] Lipinska P., Allen S.V., Hopkins W.G. (2016). Relationships between pacing parameters and performance of elite male 1500-m swimmers. Int. J. Sports Physiol. Perform..

[10] Wu S.S., Peiffer J.J., Peeling P., Brisswalter J., Lau W.Y., Nosaka K., Abbiss C.R. (2016). Improvement of sprint triathlon performance in trained athletes with positive swim pacing. Int. J. Sports Physiol. Perform..

[11] Konings M.J., Hettinga F.J. (2018). Pacing decision making in sport and the effects of interpersonal competition: A critical review. Sports Med..

[12] Micklewright D., Parry D., Robinson T., Deacon G., Renfree A., St Clair Gibson A., Matthews W.J. (2015). Risk perception influences athletic pacing strategy. Med. Sci. Sports Exerc..

[13] Schiphof-Godart L., Hettinga F.J. (2017). Passion and pacing in endurance performance. Front. Physiol..

[14] Santos-Lozano A., Collado P.S., Foster C., Lucia A., Garatachea N. (2014). Influence of sex and level on marathon pacing strategy. Insights from the New York city race. Int. J. Sports Med..

[15] Nikolaidis P.T., Knechtle B. (2017). Do fast older runners pace differently from fast younger runners in the “New York city marathon”?. J. Strength Cond. Res..

[16] Nikolaidis P.T., Knechtle B. (2018). Pacing in age group marathoners in the “New York city marathon”. Res. Sports Med..

[17] Filipas L., Nerli Ballati E., Bonato M., La Torre A., Piacentini M.F. (2018). Elite male and female 800-m runners’ display of different pacing strategies during season-best performances. Int. J. Sports Physiol. Perform..

[18] Deaner R.O., Lowen A. (2016). Males and females pace differently in high school cross-country races. J. Strength Cond. Res..

[19] Deaner R.O., Addona V., Carter R.E., Joyner M.J., Hunter S.K. (2016). Fast men slow more than fast women in a 10 kilometer road race. PeerJ.

[20] March D.S., Vanderburgh P.M., Titlebaum P.J., Hoops M.L. (2011). Age, sex, and finish time as determinants of pacing in the marathon. J. Strength Cond. Res..

[21] Renfree A., Crivoi do Carmo E., Martin L. (2016). The influence of performance level, age and gender on pacing strategy during a 100-km ultramarathon. Eur. J. Sport Sci..

[22] Bertuzzi R., Lima-Silva A.E., Pires F.O., Damasceno M.V., Bueno S., Pasqua L.A., Bishop D.J. (2014). Pacing strategy determinants during a 10-km running time trial: Contributions of perceived effort, physiological, and muscular parameters. J. Strength Cond. Res..

[23] Nikolaidis P.T., Knechtle B. (2018). Pacing strategies in the ‘Athens classic marathon’: Physiological and psychological aspects. Front. Physiol..

[24] Diaz J.J., Fernandez-Ozcorta E.J., Santos-Concejero J. (2018). The influence of pacing strategy on marathon world records. Eur. J. Sport Sci..

[25] Hanley B. (2016). Pacing, packing and sex-based differences in Olympic and IAAF world championship marathons. J. Sports Sci..

[26] Trubee N.W., Vanderburgh P.M., Diestelkamp W.S., Jackson K.J. (2014). Effects of heat stress and sex on pacing in marathon runners. J. Strength Cond. Res..

[27] http://vw-ljubljanskimaraton.si.

[28] Knechtle B., Di Gangi S., Rust C.A., Rosemann T., Nikolaidis P.T. (2018). Men’s participation and performance in the ‘Boston marathon’ from 1897 to 2017. Int. J. Sports Med..

[29] Cohen J. (1988). Statistical Power Analysis for the Behavioral Sciences.

[30] Abbiss C.R., Laursen P.B. (2008). Describing and understanding pacing strategies during athletic competition. Sports Med..

[31] Faulkner J., Parfitt G., Eston R. (2008). The rating of perceived exertion during competitive running scales with time. Psychophysiology.

[32] Deaner R.O., Carter R.E., Joyner M.J., Hunter S.K. (2014). Men are more likely than women to slow in the marathon. Med. Sci. Sports Exerc..

[33] Black M.I., Jones A.M., Bailey S.J., Vanhatalo A. (2015). Self-pacing increases critical power and improves performance during severe-intensity exercise. Appl. Physiol. Nutr. Metab..

[34] Schallig W., Veneman T., Noordhof D.A., Rodriguez-Marroyo J.A., Porcari J.P., de Koning J.J., Foster C. (2018). The role of the rating-of-perceived-exertion template in pacing. Int. J. Sports Physiol. Perform..

[35] Tomazini F., Pasqua L.A., Damasceno M.V., Silva-Cavalcante M.D., de Oliveira F.R., Lima-Silva A.E., Bertuzzi R. (2015). Head-to-head running race simulation alters pacing strategy, performance, and mood state. Physiol. Behav..

[36] Renfree A., Casado A. (2018). Athletic races represent complex systems, and pacing behavior should be viewed as an emergent phenomenon. Front. Physiol..

[37] Brick N.E., MacIntyre T.E., Campbell M.J. (2016). Thinking and action: A cognitive perspective on self-regulation during endurance performance. Front. Physiol..

[38] Knechtle B., Nikolaidis P.T. (2018). Sex- and age-related differences in half-marathon performance and competitiveness in the world’s largest half-marathon-The goteborgsvarvet. Res. Sports Med..

[39] Salinero J.J., Soriano M.L., Lara B., Gallo-Salazar C., Areces F., Ruiz-Vicente D., Abian-Vicen J., Gonzalez-Millan C., Del Coso J. (2017). Predicting race time in male amateur marathon runners. J. Sports Med. Phys. Fit..

